# Thymoma type B2 progression, due to fear of contamination, in association with hydrocephalus: A case report of avoidant behavior during COVID-19 pandemic

**DOI:** 10.1016/j.radcr.2021.12.028

**Published:** 2021-12-28

**Authors:** Aleksandar Georgiev, Atanas Hilendarov, Silvia Tsvetkova, Anna Vasilska

**Affiliations:** a Department of Diagnostic Imaging, Medical University Plovdiv, Bul. Vasil Aprilov 15A, Plovdiv, Bulgaria; b Complex Oncology Center Plovdiv, Plovdiv, Bulgaria

**Keywords:** Fear, COVID 19, Pandemics, Thymoma type B2, Oncological progression, Psychological care

## Abstract

The novel coronavirus pandemic outbreak caused extreme public fear, health concerns, and psychological distress, especially in oncology patients. The presented rare case is of a 40-year-old female with thymoma type B, with rapid progression, due to fear from the COVID-19 pandemic. Biopsy and histological verification of the tumor show a B-type thymoma with a high proliferative index. The fear of infection is causing avoidant behavior and leads to suboptimal therapy in some oncology patients that will have severe consequences. We can conclude that adequate, personalized, and most importantly, active psychological care is necessary and should be implemented for cancer patients. To be prepared for a future lockdown, it may be helpful to urge patients to seek alternative forms of social contact, such as online and mobile communications, to combat depression lockdown effects.

## Introduction

The 2019 novel coronavirus disease (COVID‐19) outbreak caused extraordinary public health concerns and tremendous psychological distress [1,2]. Especially in oncology patients. At even more significant risks are those with rare oncological diseases such as the presented case. Fear of the novel virus and postponement of treatment can have severe consequences. Histologic types of thymomas are based on morphologic features. The World Health Organization system divides thymic epithelial tumors, which are below 1% of all neoplasms, into 6 clinically relevant categories – A, AB, B1, B2, B3, and thymic carcinoma. The proportion of B2 types is around 20% for all thymomas. The classification correlates with the Masaoka staging system, which reflects the extent and invasiveness of thymomas [3–5].

## Case presentation

A rare case of a 40-year-old female patient with thymoma type B2, with a high proliferative index. Biopsy and histological verification of the tumor: pan CK AE1/AE3 - negative expression; p 63 - positive local expression; BCL - positive local expression; TTF1 - negative expression; NCAM - negative expression; CD3 - positive local expression; CD20 - positive local expression and Ki 67 ≈ 60%. The patient has congenital hydrocephalus due to enlarged back horns of lateral ventricles. She has paresis of her right leg due to the intracranial compression developed at the age of 6. The patient has normal baseline intellect and mental health. All patients are tested for COVID 19 with an antigen test before they are hospitalized.

A staging 64-slice helical CT scan with water-soluble iodine contrast media was performed with normal, sharp, and soft reconstructions. The routine CT protocol used for staging and restaging the disease include head, neck, and whole-body scan.

**Fig. 1:**
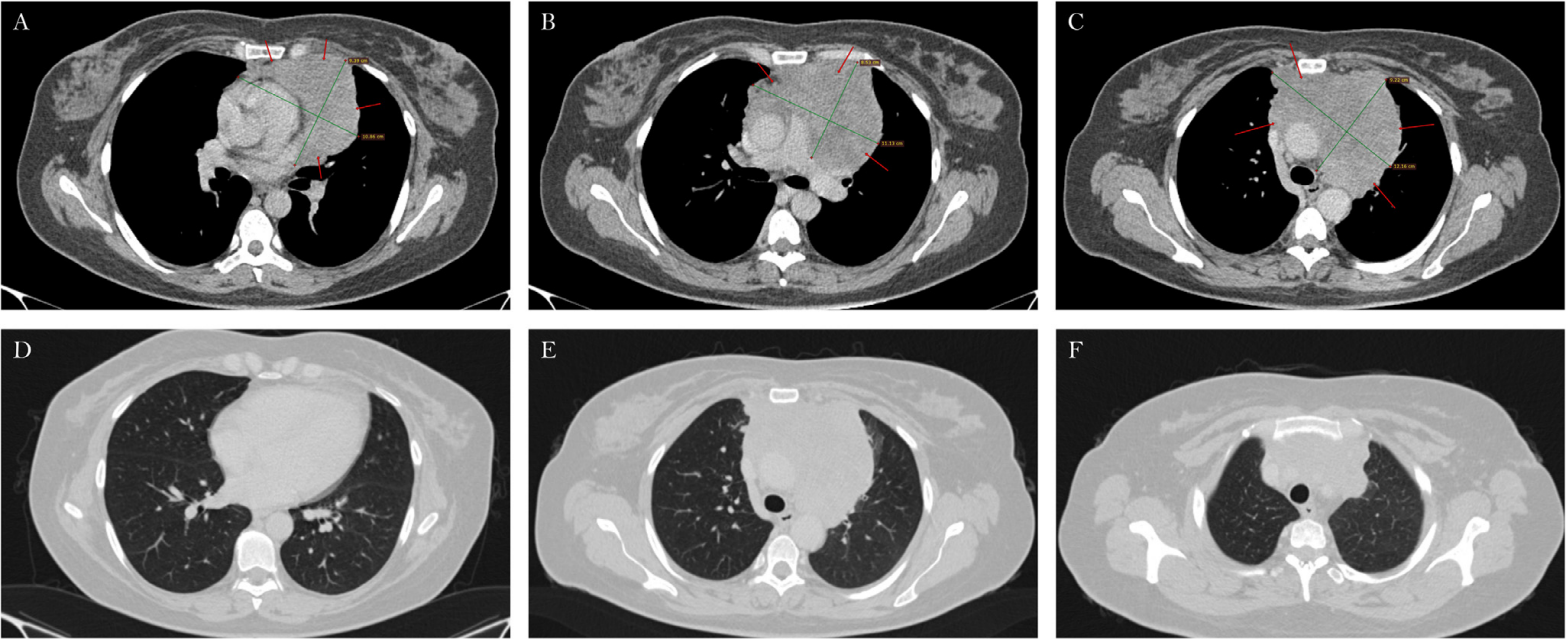
Initial CT findings in axial view. A Thymic formation with hetero-dense characteristics is visible in the mediastinum (A, B, C). There is no visible metastatic disease in the lung window.

The initial CT scan demonstrates a large thymic, heterodense formation with cystic zones. The mass surrounds the great vessels. They do not appear invaded by the tumor. There are no pleural effusions or visible metastatic disease elsewhere (Fig. 1).

In the same period, the pandemic outbreak forced governments to impose restrictive measures with the same objective - social distance and isolation. However, their impact on mental health was not recognized initially. No vaccine was available at that time period.

Three months later, due to clinical complaints from chest pain behind the sternum and difficulty breathing, another restaging CT scan was performed. Before the exam, the patient reported that she was terrified of the neoplasm, but she was even more afraid of the uncertainty and instability that the pandemic brought. The knowledge of the higher risk of infections related to thymomas further augmented her fear. Therefore, she decided not to leave her home and postpone the chemotherapy—a decision she made out of fear, as she explained.

**Fig. 2:**
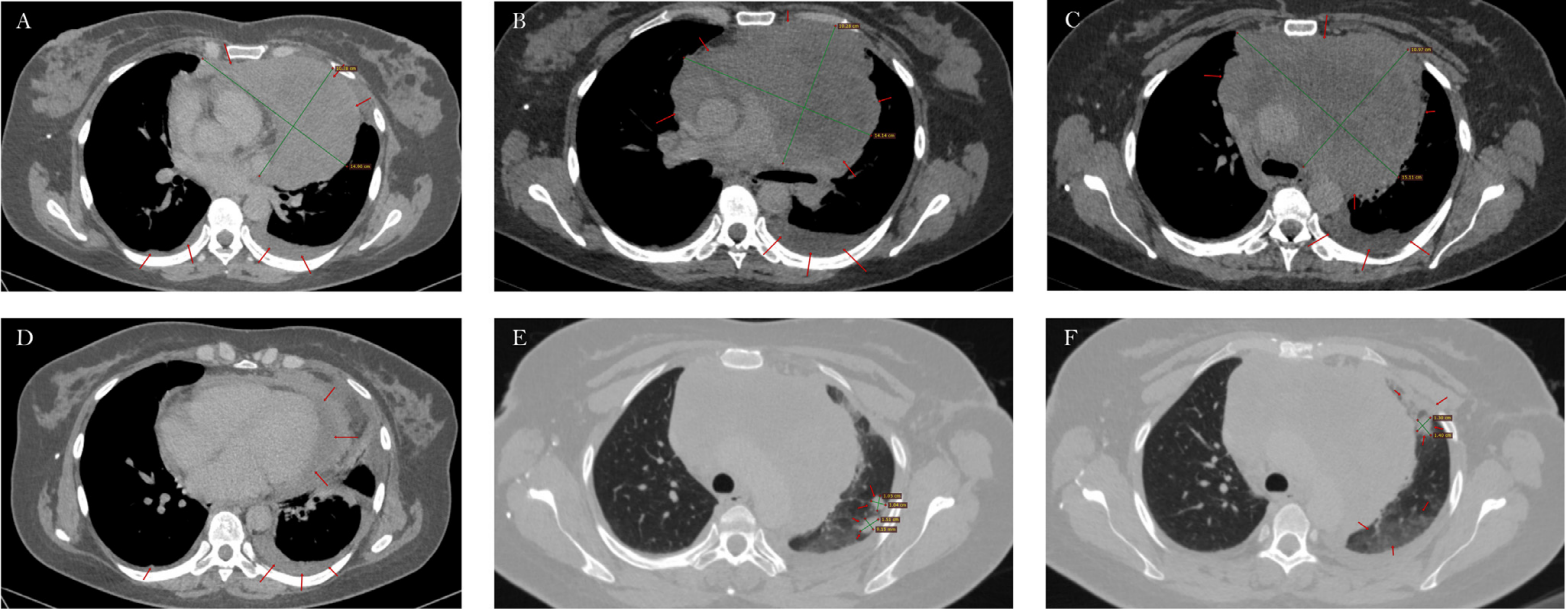
Follow-up CT scan after 3 months. Visible changes in tumor size and metastatic disease – axial view (A, B, C). Pericardial and left-sided pleural effusion.

We can see that the thymoma has enlarged by almost 50% of its original size on the second axial slice. There is visible pericardial effusion, metastatic disease in the left lung with a pleural effusion on the same side. We can observe some newly formed “ground glass” opacities probably due to hypoventilation. Thickening of the dorsal pleura on the right side is also visible (Fig. 2).

**Fig. 3:**
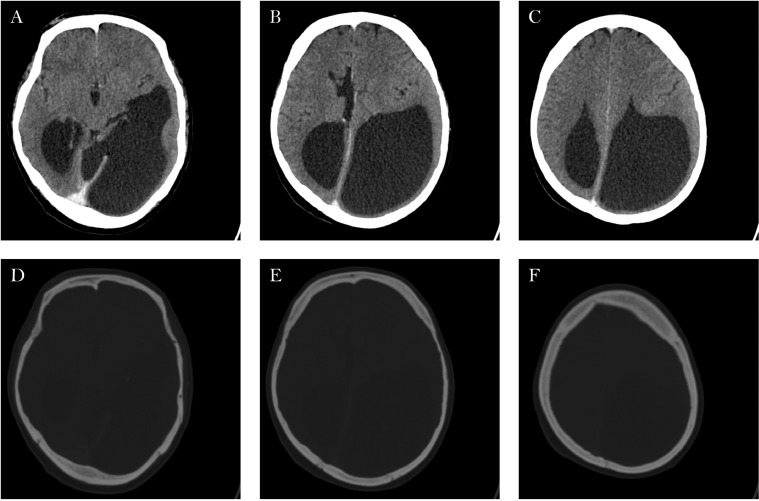
Axial CT scan reveals the enlarged ventricles (A, B, C); In the bone window, we can observe the skull's deformation (D, E, F).

All findings described above indicate a progression of the neoplastic disease from III to IVB stage according to Masaoka classification. The great vessels appear intact on the follow-up CT scan. The patient has congenital hydrocephalus due to enlarged back horns of the lateral ventricles. The skull is deformed from high intracranial pressure in infancy. Compression and dislocation of brain structures can be observed in Figure 3.

No other malformations are visible on the CT scans.

After convincing the patient to seek mental help, she started psychological consultations and began her chemotherapy treatment a week later.

## Discussion

The presented rare case of a rapid progression due to fear from the COVID 19 pandemic is unfortunate. Thymoma has a prevalence of 0.1 to 0.4/100,000 and accounts for less than 1% of all malignant tumors [1,2]. The B2 type of thymoma is defined as a tumor in which the neoplastic epithelial component appears as scattered plump cells with vesicular nuclei and distinct nucleoli among a heavy lymphocyte population by the World Health Organization classification [3]. The proportion of B2 types reported is about 20% for all thymomas. The B2 type of thymoma has been shown to have a better prognosis than thymic carcinoma and worse than types A, AB, and B1 [4,5]. Thymomas usually occur in the sixth decade of life and have no significant gender predilection. About half of the patients were staged as III or IV at the first diagnosis [3,5,6]. The Masaoka staging system is an independent prognostic factor for all thymoma subtypes in previous research [6]. The 5-year survival rate for type B2 thymoma in stage IV is about 34.3% [2]. Complications may include blood vessel damage, postoperative myasthenic crisis, pain, and a higher risk of infections.

As the COVID-19 pandemic continues to ravage populations across the world, changes in health-seeking behavior and the availability of and access to essential diagnostic services [7] are expected to lead to many deaths due to different types of cancer. Both short-term (1 year) and long-term (5 years) survival rates are expected to drop significantly. The presented rare case demonstrates avoidant behavior that leads to suboptimal treatment due to fear of COVID-19 infection. This is exacerbated by the fact that thymoma is a neoplasm that lacks optimal protocols due to the rarity of the disease [8]. Poor recognition of depression and anxiety is shown to be associated with impaired survival in cancer patients [9], [10], [11]. Cancer patients receiving early psychological interventions had less aggressive care at the end of life and more prolonged survival [12,13]. Initially, we thought oncology patients would be more worried about disease progression than the epidemiological conditions and would wish to continue chemotherapy during the COVID-19 pandemic [14,15]. Unfortunately, the presented case demonstrates another point of view– the one induced by fear of COVID-19 infection and isolation depression. During this newly emerged situation, it is essential to acknowledge the challenges this pandemic poses regarding vulnerable cancer patients’ care and the subsequent psychosocial impact [13]. The government imposed measures such as partial or complete lockdown, social distance, and media pressure all lead to serious depressive disorders. Contrary to our initial beliefs, some people are more afraid of the potential COVID-19 infection rather than the outcome of their oncological disorder. It is vital to acknowledge the existence of such people and the necessity of implementing immediate and active psychological care. To be prepared for a future lockdown, it may be helpful to urge patients to seek alternative forms of social contacts [16], such as online and mobile communications, to combat depression lockdown effects.

Patient preferences and values have been at the center of modern medicine. However, at the end of the day, patients rely heavily on our medical expertise in formulating the optimal treatment [13].

## Conclusions

Fear of Covid-19 infection causes avoidant patient behavior and overall suboptimal oncological treatment that will have severe consequences. Adequate, personalized, and most importantly, active psychological care is necessary and should be implemented among affected cancer patients.

## Patient consent

Patient consent has been obtained.
